# Acute Kidney Injury Increase Risk of Left Ventricular Remodeling: A Cohort of 1,573 Patients

**DOI:** 10.3389/fphys.2021.744735

**Published:** 2021-09-27

**Authors:** Qiang Li, Weihua Chen, Shanshan Shi, Haozhang Huang, Wenguang Lai, Liwei Liu, Ming Ying, Bo Wang, Huanqiang Li, Zhidong Huang, Liling Chen, Jiyan Chen, Shiqun Chen, Jin Liu, Yong Liu

**Affiliations:** ^1^Guangdong Provincial Key Laboratory of Coronary Heart Disease Prevention, Department of Cardiology, Guangdong Cardiovascular Institute, Guangdong Provincial People’s Hospital, Guangdong Academy of Medical Sciences, Guangzhou, China; ^2^The School of Clinical Medicine, Fujian Medical University, Fuzhou, China; ^3^The Second School of Clinical Medicine, Southern Medical University, Guangzhou, China; ^4^Guangdong Provincial People’s Hospital, School of Medicine, South China University of Technology, Guangzhou, China; ^5^Longyan First Affiliated Hospital of Fujian Medical University, Longyan, China

**Keywords:** coronary angiography, incidence, cardiac remodeling, acute kidney injury, left ventricular remodeling

## Abstract

**Background:** Acute kidney injury (AKI) is a common complication after coronary angiography (CAG) and associated with heart failure (HF). Left ventricular (LV) remodeling is a vital process in the progression of HF. However, few studies investigate the relationship between AKI and LV remodeling.

**Methods:** We included consecutive patients undergoing CAG from January 2007 to December 2018 at Guangdong Provincial People’s Hospital (NCT04407936). AKI was defined as an absolute increase in serum creatinine (Scr) of ≥ 0.3mg/dl or a ≥ 50% increase in Scr from baseline within the first 48–72 h after the procedure. LV remodeling was defined as: (1) an absolute decrease in left ventricular ejection fraction (LVEF) of ≥ 10% compared to baseline, or (2) a follow-up LVEF < 40%. Univariate and multivariate logistical regressions were used to assess the association between AKI and LV remodeling.

**Results:** Of the 1,573 patients (62.2 ± 9.7 years, female 36.7%) included in the study, 231 (14.7%) had AKI. The incidence of LV remodeling was higher in patients with AKI than in those without AKI (24.7% vs. 14.5%). After adjusting for confounding, multivariate logistic regression showed that AKI was associated with a significantly higher risk of LV remodeling [adjusted odds ratio (aOR) 1.87; 95% CI, 1.30–2.66; *p* < 0.001]. In addition, LV remodeling patients had higher all-cause mortality compared to non-LV remodeling patients (9.7% vs. 19.1%).

**Conclusion:** Our data suggested that AKI is present in up to 15% of patients after CAG and that nearly a quarter of AKI patients suffered LV remodeling and AKI patients have a two-fold risk of developing LV remodeling than non-AKI patients. Our findings suggest that more active measures be taken not only to prevent AKI patient developing into LV remodeling, but to prevent patients undergoing CAG from developing AKI.

## Introduction

Acute kidney injury (AKI) is a common complication after coronary angiography (CAG) and interventional procedures, with an estimated incidence of up to 12% (Lun et al., 2021).

Previous studies have shown that AKI is strongly associated with the development of heart failure (HF). Odutayo A’s meta-analysis showed that AKI was associated with a 58% increased risk of HF (Odutayo et al., 2017). Studies also found that the patient with AKI had higher levels of oxidative stress, inflammation, calcium transport abnormalities, and other pathophysiological mechanisms through activation of the renin-angiotensin system (Robinson et al., 1992; Alge et al., 2013a; Ni et al., 2018). In addition, oxidative stress, inflammatory responses, calcium transport abnormalities and especially the renin-angiotensin system also play an essential part in the pathophysiology of left ventricular (LV) remodeling (Azevedo et al., 2016). However, it remains unclear whether AKI is independently associated with LV remodeling. LV remodeling is one of the most important processes in the occurrence and development of HF. Early diagnosis and treatment can further reduce the development to HF.

The study aims to investigate the association between AKI and LV remodeling in a large Chinese population who underwent CAG. On this basis, clinicians can be guided to identify and intervene early in LV remodeling and reduce the incidence of HF after AKI.

## Materials and Methods

### Study Population

**FIGURE 1:**
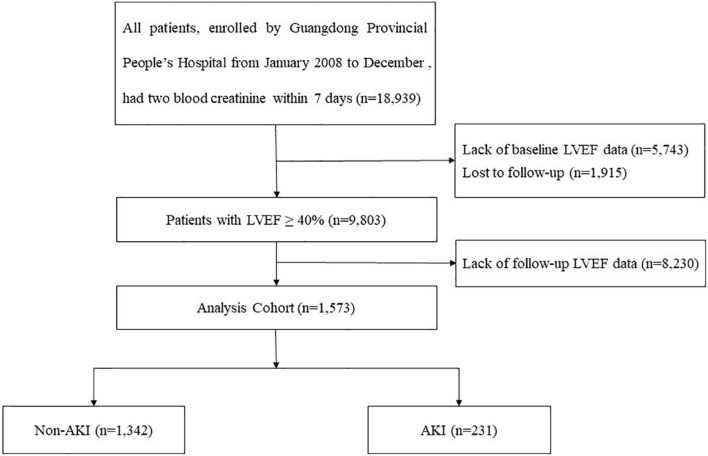
Flow chart of the study population.

We obtain data from the registry of Cardiorenal Improvement (CIN) study (ClinicalTrials.gov; NCT04407936) from January 2007 to December 2018 at Guangdong Provincial People’s Hospital. A total of 1,573 CAG patients were included in the final analysis after excluding patients combined with conditions as follow: (a) LVEF ≤ 40% at baseline; (b) follow-up echocardiography was not acquired within 3–12 mouths; (c) lost serum creatinine (Scr) before and after surgery (Figure 1). The cohort were divided into AKI and non-AKI groups on the basis of Scr changes. This study was conducted in accordance with the Declaration of Helsinki and was approved by the Research Ethics Committee of Guangdong Provincial People’s Hospital (No. GDREC2019555H). CAG or percutaneous coronary intervention (PCI) was performed in accordance with standard clinical practice guidelines (Kushner et al., 2009; Jneid et al., 2012; Levine et al., 2016).

### Clinical Data and Measurement

In this study, we retrieved all of clinical data of 1,573 patients during their first hospitalization from data base of Guangdong Provincial People’s Hospital, including demographic characteristics, medical comorbidities, laboratory examinations, echocardiography and medications at discharge. Echocardiography was performed by a trained cardiac ultrasound doctor for all patients at the time of admission. The calculations for LVEF used the biplane-Simpson method by the end diastolic and end systolic apical 4- and 2- chamber views. Follow-up echocardiography was assessed over 3–12 months after hospitalization by the same method, the median follow-up echocardiography time was 3.5 months.

### Definition of Deterioration and Outcomes

The primary endpoint was LV remodeling, defined as: (1) an absolute decrease in LVEF of ≥ 10% compared to baseline, or (2) a follow-up LVEF < 40% (Puls et al., 2020). AKI was defined as an absolute Scr increase ≥ 0.3 mg/dl or a relative increase in Scr ≥ 50% within 48 h after contrast-medium exposure (van der Molen et al., 2018). Estimated glomerular filtration rate (eGFR) was estimated by the Modification of Diet in Renal Disease (MDRD) formula, and chronic kidney disease (CKD) was defined as eGFR < 60 ml/min/1.73 m^2^ (Levey et al., 1999). Congestive heart failure (CHF) was defined as New York Heart Association (NYHA) functional class > 2, Killip class > 1 or pulmonary edema.

### Statistical Analysis

The study population was divided into two groups AKI and non-AKI. Data was presented as the mean with standard deviation (SD) or median with interquartile range (IQR) for continuous variables and quantity and frequency (%) for categorical variables. Categorical variables were compared by Pearson chi-squared test, and continuous variables by *t*-test. The association between AKI and LV remodeling was tested by univariable and multivariable logistic regression. Model 1 was unadjusted, Model 2 was adjusted with demographic characteristics (age and gender), and Model 3 was adjusted with medical comorbidities and treatment based on Model 2 [acute myocardial infarction (AMI), PCI, diabetes (DM), hypertension (HT), CKD, CHF, angiotensin-converting enzyme inhibitor/angiotensin receptor blocker (ACEI/ARB) and contrast medium volume (CMV)]. All analyses were performed by R software (version 4.0.3; R Foundation for Statistical Computing, Vienna, Austria). A two-sided *P-*value < 0.05 indicated the significance for all analyses.

## Results

### Clinical and Procedural Characteristics

The patient population selected for the study consisted of 1,573 patients, of whom 997 were male and 576 were females (mean age 62.2 ± 9.7 years). In total, 930 (59.1%) had coronary artery disease (CAD), 367 (23.4%) had DM, 313 (19.9%) had CHF and 438 (27.8%) had CKD. The population was divided into 2 groups subsequently: AKI (*n* = 231) and non-AKI (*n* = 1,342). Compared to the non-AKI patients, those in the AKI group had higher pro-brain natriuretic peptide (pro-BNP), a larger left ventricular end-diastolic dimension (LVEDD), left ventricular end-systolic dimension (LVESD) and more likely combined with CHF. On the contrast, the AKI group was less likely to be combined with DM, HT, and had fewer uses of angiotensin-converting enzyme inhibitor/angiotensin receptor blocker (ACEI/ARB). The detailed patients’ clinical characteristics are listed in Table 1.

**TABLE 1:** Baseline Characteristics According to Categories of AKI.

Characteristic	Overall	Non-AKI	AKI	*P*-value
	(*N* = 1,573)	(*N* = 1,342)	(*N* = 231)	
**Demographic**				
Age, years	62.23 (9.74)	62.37 (9.75)	61.45 (9.69)	0.185
Age > 75, n (%)	145 (9.22)	127 (9.46)	18 (7.79)	0.491
Female, n (%)	576 (36.62)	476 (35.47)	100 (43.29)	0.027
**Medical history**				
AMI, n (%)	249 (15.84)	235 (17.52)	14 (6.06)	<0.001
CAD, n (%)	930 (59.12)	846 (63.04)	84 (36.36)	<0.001
HT, n (%)	747 (47.52)	660 (49.22)	87 (37.66)	0.001
DM, n (%)	367 (23.35)	330 (24.61)	37 (16.02)	0.006
CKD, n (%)	438 (27.84)	371 (27.65)	67 (29.00)	0.729
CHF, n (%)	313 (19.91)	247 (18.42)	66 (28.57)	0.001
PCI, n (%)	699 (44.44)	650 (48.44)	49 (21.21)	<0.001
**Laboratory test**				
GLU, mmol/L	6.62 (3.19)	6.77 (3.29)	5.87 (2.55)	<0.001
HbA1c,%	6.34 (1.26)	6.39 (1.30)	6.04 (0.99)	<0.001
LDL-C, mmol/L	2.92 (0.97)	2.92 (0.99)	2.93 (0.86)	0.945
HDL-C, mmol/L	1.02 (0.28)	1.01 (0.28)	1.07 (0.28)	0.007
HGB, g/L	129.33 (18.84)	129.68 (18.46)	127.31 (20.86)	0.078
eGFR, ml/min/1.73 m^2^	73.24 (25.80)	73.42 (25.66)	72.21 (26.64)	0.511
pro-BNP (pg/ml, median [IQR]	688.80[177.10, 941.00]	598.05[154.60, 821.25]	1121.00[478.90, 158.00]	<0.001
**Echocardiography**				
LVEF,%	60.79 (9.04)	60.88 (9.10)	60.24 (8.64)	0.319
LVEF (Follow up), %	59.76 (9.92)	60.12 (9.53)	57.67 (11.72)	0.001
LVEDD, mm	49.12 (7.43)	48.89 (7.11)	50.51 (8.95)	0.002
LVESD, mm	32.00 (7.05)	31.79 (6.89)	33.24 (7.85)	0.004
LVPW, mm	10.11 (2.01)	10.09 (2.01)	10.20 (1.99)	0.460
IVS, mm	10.71 (2.22)	10.69 (2.24)	10.84 (2.10)	0.340
**Medication**				
ACEI/ARB, n (%)	486 (31.31)	445 (33.56)	41 (18.14)	<0.001
Beta-blockers, n (%)	1043 (67.20)	914 (68.93)	129 (57.08)	0.001
Statins, n (%)	920 (59.28)	848 (63.95)	72 (31.86)	<0.001
CCB, n (%)	327 (21.07)	284 (21.42)	43 (19.03)	0.467
**Events**				
LV remodeling, n (%)	252 (16.02)	195 (14.53)	57 (24.68)	<0.001
1 year of death, n (%)	45 (2.86)	35 (2.61)	10 (4.33)	0.217
Follow-up death, n (%)	177 (11.25)	141 (10.51)	36 (15.58)	0.032

On the basis of the diagnosis of LV remodeling, 252 patients (16.0%) had LV remodeling, the patients developed LV remodeling were elder (62.0 ± 9.8 vs. 63.4 ± 9.4, *p* = 0.04), more likely to combine CHF (18.9% vs. 25.1%, *p* = 0.03), and had lower eGFR (73.8 vs. 70.1 ml/min/1.73 m^2^, *p* = 0.04). Demographics, risk factors, and clinical and laboratory characteristics of the 1,573 patients according to the presence of LV remodeling are summarized in Supplementary Table 1.

### Incidence of Ventricular Remodeling

**FIGURE 2:**
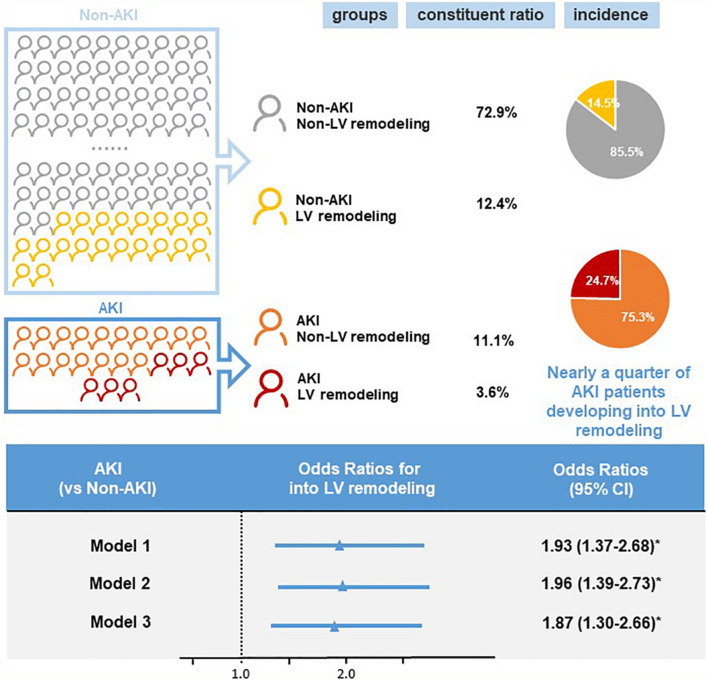
Incidence and risk of left ventricular (LV) remodeling by acute kidney injury (AKI) status. Logistic regression was used to calculate the odds ratios (OR) and 95% confidence intervals (CI) for left ventricular remodeling. Model 1: unadjusted odds ratios for acute kidney injury. Model 2: odds ratios adjusted for age and gender. Model 3: odds ratios adjusted for multiple variables (age, gender, acute myocardial infarction, percutaneous coronary intervention, diabetes, hypertension, chronic kidney disease, angiotensin-converting enzyme inhibitor/angiotensin receptor blocker, contrast medium volume). *p < 0.05.

**FIGURE 3:**
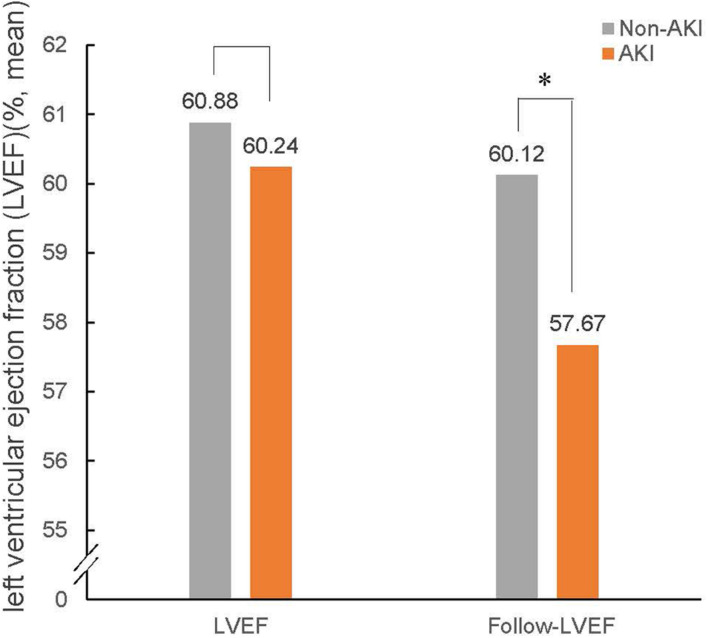
LVEF at baseline and follow-up in patients with non-AKI and AKI. The bar graphs show LVEF at baseline and follow-up in patients with non-AKI (gray) and AKI (orange). AKI, acute kidney injury; LVEF, left ventricular ejection fraction. ^∗^*P* < 0.05.

Follow-up echocardiography data showed that there were 252 cases of LV remodeling (16.0%), 57 cases of AKI (3.6%), and 195 cases of non-AKI (12.4%). Among patients with AKI, the incidence of LV remodeling was 24.7%, accounting for nearly a quarter of AKI patients. In contrast, the incidence of LV remodeling was only 14.5% in non-AKI patients (Figure 2). And the baseline LVEF results showed no significant difference between the patient with AKI and non-AKI (60.24% vs. 60.88%, *p* > 0.05), while during the 1-year follow-up, the reduction in LVEF was more pronounced in the patient with AKI compared to non-AKI (LVEF: 57.67% in AKI vs. 60.12% in non-AKI, *p* < 0.05), and the difference was statistically significant (Figure 3).

### Association Between Acute Kidney Injury and Ventricular Remodeling

**FIGURE 4:**
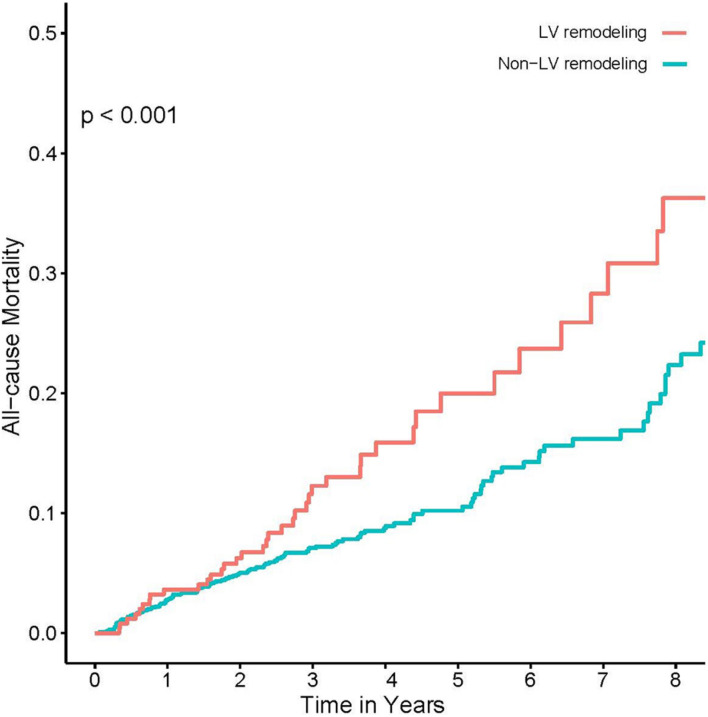
Kaplan-Meier curves for long-term all-cause mortality.

Univariate logistic regression analysis showed a significant correlation between LV remodeling and AKI [odds ratio (OR), 1.93; 95% CI, 1.37–2.68; *p* < 0.001]. After adjusting age and gender, there was still a significant correlation between LV remodeling and AKI [adjusted odds ratio (a OR) 1.96; 95% CI, 1.39–2.73; *p* < 0.001]. After adjusting demographic characteristics, medical comorbidities and treatment (age, gender, AMI, PCI, DM, HT, CKD, ACEI/ARB, and CMV) (Figure 2), LV remodeling remained significantly associated with AKI [adjusted odds ratio (a OR) 1.87; 95% CI, 1.30–2.66; *p* < 0.001]. In addition, Kaplan-Meier curves showed that LV remodeling patients had higher all-cause mortality compared to non-LV remodeling patients (Log-rank test, *p* < 0.001) (Figure 4).

## Discussion

To the best of our knowledge, this is the first study aiming to investigate the association between AKI and LV remodeling as well as to assess the incidence of AKI combined with LV remodeling and changes in related indicators. According to our results, AKI is an independent risk factor for the development of LV remodeling, and patients with LV remodeling after AKI may have a worse clinical outcome. Therefore, it is necessary to pay attention to the occurrence of LV remodeling in AKI patients, early identification and intervention, and prevention of adverse clinical outcomes.

LV remodeling is a vital process in the progression of HF, and it is defined as genomic expression, molecular, cellular and interstitial changes that clinically manifest as changes in heart size, shape and function after cardiac injury (Cohn et al., 2000). Current thinking is that LV remodeling confers short-term benefits. However, neglect can lead to maladaptation of these remodeling events and increase cardiovascular morbidity and mortality. HF has been thought to be causally related to LV remodeling. Recent studies have found that except for HF, a number of other cardiovascular diseases are also associated with LV remodeling, such as AMI, HT, valvular disease, and hypertrophic cardiomyopathy (Pfeffer and Braunwald, 1990; Harris et al., 2006; Berk et al., 2007; Chin et al., 2017). Furthermore, a growing number of studies have shown that LV remodeling is associated not only with cardiovascular disease, but also with other diseases. Alpert et al. (2018) suggest that obesity produces hemodynamic changes can cause LV remodeling. In another article on LV remodeling, Jia et al. (2016) concluded that DM mellitus is also a risk factor for LV remodeling In a clinical trial, Bajaj et al. (2020) demonstrated that CKD is associated with the development of LV remodeling and is mediated by coronary microvascular dysfunction Similarly, our study was the first to connect AKI with LV remodeling and identified AKI as an independent predictor of the occurrence of LV remodeling.

There are several possible mechanisms that could explain the link between AKI and the development of LV remodeling. First, AKI causes sustained oxidative stress and promotes dysregulation of the renin-angiotensin-aldosterone system, which induces macrophage infiltration, cardiac inflammation, and myocardial fibrosis, and leads to endothelial dysfunction and cardiac fibrosis as well as ventricular dysfunction. Induction of this system is a hallmark of the cardiovascular response to AKI (Alge et al., 2013a, b; Crowley and Rudemiller, 2017). Second, oxidative stress appears to play an important pathophysiological role in LV remodeling, and the high oxidative stress state of AKI may contribute to the occurrence of LV remodeling after AKI (Azevedo et al., 2016; Ni et al., 2018). Third, AKI causes changes in the dihydropyridine receptors on L-type calcium channels, leading to distortions in intracellular calcium homeostasis. In turn, alterations in the proteins that transport calcium may lead to cardiac dysfunction in the remodeling heart (Luo and Anderson, 2013; Feridooni et al., 2015; Burtscher et al., 2020). Fourth, deleterious upregulation of the renin-angiotensin-aldosterone system resulting in fluid retention and peripheral arterial vasoconstriction, thereby increasing the preload and afterload of the left ventricle, which is one of the important mechanisms leading to ventricular remodeling (Murphy et al., 2020).

This study had several important clinical significances and research implications. Our results demonstrated that AKI was an independent predictor of LV remodeling among patients undergoing CAG. It is estimated that there are approximately 2 million cases of hospitalized AKI each year (Goldstein et al., 2013). Based on the disparities observed in our study, this translates to approximately 50,000 AKI patients per year with combined LV remodeling, and they have a much higher mortality rate than those who do not. Therefore, this reminds clinicians that ancillary tests related to the assessment of cardiovascular disease, such as cardiac ultrasound, deserve equal attention in the follow-up of patients with AKI. Routine measurements such as LVEF can provide clinicians with useful information to identify patients at high risk for LV remodeling after AKI and thus provide more attention and prevention.

### Limitations

Our study has some limitations. First, our study used only an LVEF decrease greater than 10% for the determination of LV remodeling, which has different criteria for determination. However, an LVEF decrease greater than 10% still proved to be a good proxy for LV remodeling. Second, our study did not investigate the effect of recovery after the occurrence of AKI on LV remodeling. However, our study still demonstrates that whenever AKI occurs, there is a significantly higher probability of LV remodeling in patients with a poor prognosis. Third, patients with AKI tend to be hypervolemic, a state that could have a negative impact on the LV. Due to the lack of echocardiography examination during AKI/at discharge, our study could not confirm whether AKI patients were hypervolemic at the time. This will be improved in our future study designs. Finally, despite the use of rigorous statistical methods, there is a possibility of residual confounding, and the observational nature of this study precludes definitive conclusions about causality. It is necessary for clinicians to identify and intervene in LV remodeling early to reduce the incidence of HF after AKI.

## Conclusion

Our data suggested that AKI is present in up to 15% of patients after CAG and that nearly a quarter of AKI patients suffered LV remodeling and AKI patients have a twofold risk of LV remodeling than non-AKI patients. Additionally, LV remodeling patients had higher mortality compared to non-LV remodeling patients. It is necessary for clinicians to identify and intervene in LV remodeling early to reduce the occurrence of HF after AKI, and more importantly, to prevent patients undergoing CAG from developing AKI.

## Data Availability Statement

The data analyzed in this study is subject to the following licenses/restrictions: The datasets used and/or analyzed during the current study are available from the corresponding author on reasonable request. Requests to access these datasets should be directed to YL, liuyong@gdph.org.cn.

## Ethics Statement

The studies involving human participants were reviewed and approved by the Guangdong Provincial People’s Hospital Ethics Committee. Written informed consent for participation was not required for this study in accordance with the national legislation and the institutional requirements.

## Author Contributions

YL, JL, SC, and JC: research idea and study design. QL, WC, SS, HH, WL, LL, MY, BW, HL, ZH, and LC: data acquisition. JL and YL: data analysis and interpretation. SC and QL: statistical analysis. YL, JL, SC, and JC: supervision and mentorship. All authors contributed important intellectual content during manuscript drafting or revision and accepts accountability for the overall work by ensuring that questions pertaining to the accuracy and integrity of any portion of the work are appropriately investigated and resolved.

## Conflict of Interest

The authors declare that the research was conducted in the absence of any commercial or financial relationships that could be construed as a potential conflict of interest.

## Publisher’s Note

All claims expressed in this article are solely those of the authors and do not necessarily represent those of their affiliated organizations, or those of the publisher, the editors and the reviewers. Any product that may be evaluated in this article, or claim that may be made by its manufacturer, is not guaranteed or endorsed by the publisher.

## Abbreviations

AKI: acute kidney injury
AMI: acute myocardial infarction
CAD: coronary artery disease
HT: hypertension
DM: diabetes
CKD: chronic kidney disease
CHF: congestive heart failure
PCI: percutaneous coronary intervention
GLU: glucose
HbA1c: hemoglobin A1c
LDL-C: low-density lipoprotein cholesterol
HDL-C: high-density lipoprotein cholesterol
HGB: hemoglobin
eGFR: estimated glomerular filtration rate
pro-BNP: pro-brain natriuretic peptide
LVEF: left ventricular ejection fraction
LVEDD: left ventricular end-diastolic dimension
LVESD: left ventricular end-systolic dimension
LVPW: left ventricular posterior wall
IVS: interventricular septal thickness
ACEI/ARB: angiotensin-converting enzyme inhibitor/angiotensin receptor blocker
CCB: calcium channel blockers
LV: left ventricular.
