# Supplementary material for: Acute Kidney Injury Increase Risk of Left Ventricular Remodeling: A Cohort of 1,573 Patients
Source: Front Physiol. 2021 Sep 27;12:744735. doi: 10.3389/fphys.2021.744735 (PMC8502848; doi:10.3389/fphys.2021.744735)
Supplement: Supplementary file 1 [file Data_Sheet_1.pdf]

SUPPLEMENTAL MATERIAL.

Supplementary Table I. Baseline Characteristics According to Categories of LV remodeling

Characteristic	Overall (N=1,573)	Non-LV remodeling (N=1,321)	LV remodeling (N=252)	P-value
**Demographic**				
Age, years	62.23 (9.74)	62.02 (9.79)	63.38 (9.41)	0.042
Age>75, n (%)	145 (9.22)	116 (8.78)	29 (11.51)	0.210
Female, n (%)	576 (36.62)	481 (36.41)	95 (37.70)	0.751
**Medical history**				
AMI, n (%)	249 (15.84)	216 (16.35)	33 (13.15)	0.238
CAD, n (%)	930 (59.12)	774 (58.59)	156 (61.90)	0.363
HT, n (%)	747 (47.52)	619 (46.86)	128 (51.00)	0.257
DM, n (%)	367 (23.35)	301 (22.79)	66 (26.29)	0.261
CKD, n (%)	438 (27.84)	363 (27.48)	75 (29.76)	0.507
CHF, n (%)	313 (19.91)	250 (18.93)	63 (25.10)	0.031
PCI, n (%)	699 (44.44)	595 (45.04)	104 (41.27)	0.301
**Laboratory test**				
GLU, mmol/L	6.62 (3.19)	6.61 (3.30)	6.64 (2.51)	0.909
HbA1c, %	6.34 (1.26)	6.32 (1.24)	6.40 (1.35)	0.456
LDL-C, mmol/L	2.92 (0.97)	2.95 (0.98)	2.81 (0.93)	0.042
HDL-C, mmol/L	1.02 (0.28)	1.03 (0.29)	0.99 (0.27)	0.042
HGB, g/L	129.33 (18.84)	129.61 (18.87)	127.87 (18.68)	0.180

eGFR, ml/min/1.73 m^2	73.24 (25.80)	73.83 (26.23)	70.14 (23.24)	0.037
pro-BNP (pg/ml, median [IQR]	688.80 [177.10, 941.00]	580.60 [157.73,1768.25]	1267.00 [433.30, 974.00]	<0.001
**Echocardiography**				
LVEF, %	60.79 (9.04)	60.51 (8.33)	62.24 (12.00)	0.005
LVEF (Follow up), %	59.76 (9.92)	62.24 (7.19)	46.73 (11.86)	<0.001
LVEDD, mm LVESD,	49.12 (7.43)	48.51 (6.89)	52.35 (9.12)	<0.001
mm	32.00 (7.05)	31.60 (6.56)	34.13 (8.96)	<0.001
LVPW, mm	10.11 (2.01)	10.11 (1.81)	10.09 (2.82)	0.887
IVS, mm	10.71 (2.22)	10.72 (2.18)	10.68 (2.44)	0.829
**Medication**				
ACEI/ARB, n (%) Beta-	486 (31.31)	404 (30.96)	82 (33.20)	0.534
blockers, n (%) Statins, n	1043 (67.20)	870 (66.67)	173 (70.04)	0.336
(%)	920 (59.28)	777 (59.54)	143 (57.89)	0.680
CCB, n (%)	327 (21.07)	285 (21.84)	42 (17.00)	0.104
**Events**				
1 year of death, n (%)	45 (2.86)	36 (2.73)	9 (3.57)	0.595
Follow-up death, n (%)	177 (11.25)	129 (9.77)	48 (19.05)	<0.001

Abbreviation: LV, left ventricular; AMI, acute myocardial infarction; CAD, coronary artery disease; HT, hypertension; DM, diabetes; CKD, chronic kidney disease; CHF, congestive heart failure; PCI, percutaneous coronary intervention; GLU, glucose; HbA1c, hemoglobin A1c; LDL-C, low-density lipoprotein cholesterol; HDL-C, high-density lipoprotein cholesterol; HGB, hemoglobin; eGFR, estimated glomerular filtration rate;

pro-BNP, pro-brain natriuretic peptide; LVEF, left ventricular ejection fraction; LVEDD, left ventricular end-diastolic dimension; LVESD, left ventricular end-systolic dimension; LVPW, left ventricular posterior wall; IVS, interventricular septal thickness; ACEI/ARB, angiotensin-converting enzyme inhibitor/angiotensin receptor blocker; CCB, calcium channel blockers.
